# Supplementary material for: Carbon information disclosure and corporate financial performance—Empirical evidence based on heavily polluting industries in China
Source: PLoS One. 2025 Jan 17;20(1):e0313638. doi: 10.1371/journal.pone.0313638 (PMC11741645; doi:10.1371/journal.pone.0313638)
Supplement: S1 Data — (ZIP) [file pone.0313638.s001.zip › DATA/Table/Correlation analysis.rtf]

Roa w	Score w	Tang w	SOEs	Size2 w	Grow~1 w	Intang w		
Roa w	1							
Score w	0.086***	1						
Tang w	-0.191***	0.065***	1					
SOEs	-0.214***	0.079***	0.298***	1				
Size2 w	0.064***	0.362***	0.271***	0.315***	1			
Growth1 w	0.056**	-0.038*	-0.130***	0.00400	-0.145***	1		
Intang w	0.00200	0.070***	-0.240***	0.062***	0.079***	0.0350	1	
Invent w	-0.089***	-0.0100	0.179***	-0.114***	-0.0100	0.0270	-0.143***	
Ltd w	-0.217***	0.144***	0.292***	0.290***	0.281***	-0.043**	-0.0220	
								
|Invent w	Ltd w							
Invent w	1							
Ltd w	-0.338***	1						
