# Supplementary material for: Carbon information disclosure and corporate financial performance—Empirical evidence based on heavily polluting industries in China
Source: PLoS One. 2025 Jan 17;20(1):e0313638. doi: 10.1371/journal.pone.0313638 (PMC11741645; doi:10.1371/journal.pone.0313638)
Supplement: S1 Data — (ZIP) [file pone.0313638.s001.zip › DATA/Table/Descriptive statistics.rtf]

Variable	N	Mean	SD	Min	p25	p50	p75	Max	
Roa w	2084	0.0360	0.0550	-0.204	0.0110	0.0320	0.0630	0.204	
Score w	2084	0.0900	0.0820	0.00300	0.0290	0.0630	0.126	0.400	
Tang w	2084	0.470	0.162	0.0680	0.354	0.475	0.589	0.796	
SOEs	2084	0.612	0.487	0	0	1	1	1	
Size2 w	2084	22.93	1.514	18.91	21.71	22.89	24.01	25.92	
Growth1 w	2084	0.0920	0.405	-0.684	-0.0810	0.0350	0.179	3.816	
Intang w	2084	0.0550	0.0520	0.00100	0.0240	0.0400	0.0660	0.339	
Invent w	2084	0.103	0.0880	0.00100	0.0380	0.0850	0.141	0.408	
Ltd w	2084	0.138	0.122	0	0.0410	0.109	0.198	0.505	
