# Supplementary material for: Carbon information disclosure and corporate financial performance—Empirical evidence based on heavily polluting industries in China
Source: PLoS One. 2025 Jan 17;20(1):e0313638. doi: 10.1371/journal.pone.0313638 (PMC11741645; doi:10.1371/journal.pone.0313638)
Supplement: S1 Data — (ZIP) [file pone.0313638.s001.zip › DATA/Table/Instrumental variables regression results.doc]

	(1)	(2)	
	firstScore_w	second	
VARIABLES	Score_w	Roa_w	
			
tool	0.837***		
	(16.35)		
Tang_w	0.007	-0.034***	
	(0.58)	(-3.77)	
SOEs	-0.008**	-0.024***	
	(-2.06)	(-8.13)	
Size2_w	0.015***	0.008***	
	(11.22)	(5.94)	
Growth1_w	0.003	0.010***	
	(0.96)	(3.37)	
Intang_w	0.026	-0.003	
	(0.65)	(-0.10)	
Invent_w	0.036*	-0.102***	
	(1.65)	(-6.44)	
Ltd_w	0.012	-0.160***	
	(0.65)	(-12.39)	
YEAR1	-0.001	0.012**	
	(-0.15)	(2.28)	
YEAR2	0.001	0.005	
	(0.14)	(0.84)	
YEAR3	-0.000	-0.009	
	(-0.02)	(-1.50)	
YEAR4	-0.001	0.001	
	(-0.07)	(0.12)	
YEAR5	-0.004	0.011**	
	(-0.36)	(2.06)	
YEAR6	-0.006	0.011*	
	(-0.66)	(1.87)	
YEAR7	-0.004	0.010**	
	(-0.36)	(1.97)	
YEAR8	-0.004	0.006	
	(-0.45)	(1.19)	
YEAR9	-0.002	0.020***	
	(-0.27)	(4.00)	
YEAR10	-0.005	0.004	
	(-0.49)	(0.72)	
o.YEAR11	-	-	
			
INDUS1	0.007	-0.033***	
	(0.96)	(-6.50)	
INDUS2	-0.011	-0.014*	
	(-1.33)	(-1.90)	
INDUS3	-0.004	-0.026***	
	(-0.68)	(-5.86)	
INDUS4	-0.007	-0.029***	
	(-1.03)	(-6.25)	
o.INDUS5	-	-	
			
Score_w		0.136***	
		(3.01)	
Constant	-0.330***	-0.072***	
	(-11.35)	(-2.86)	
			
Observations	2,084	2,084	
R-squared		0.188	
Robust t-statistics in parentheses
*** p<0.01, ** p<0.05, * p<0.1
