# Supplementary material for: Carbon information disclosure and corporate financial performance—Empirical evidence based on heavily polluting industries in China
Source: PLoS One. 2025 Jan 17;20(1):e0313638. doi: 10.1371/journal.pone.0313638 (PMC11741645; doi:10.1371/journal.pone.0313638)
Supplement: Attachment — Submitted filename: PONE-D-24-32603_reviewer.pdf [file pone.0313638.s002.pdf]

PLOS ONE

Carbon information disclosure and corporate financial performance— Empirical evidence based on heavily polluting industries in China

--Manuscript Draft--

**Manuscript Number:**	PONE-D-24-32603
**Article Type:**	Research Article
**Full Title:**	Carbon information disclosure and corporate financial performance— Empirical evidence based on heavily polluting industries in China
**Short Title:**	Carbon information disclosure and corporate financial performance
**Corresponding Author:**	Yuan Yuan Su<br>Huaqiao University<br>Quanzhou, CHINA
**Keywords:**	Carbon disclosure; institutional investors; cost of debt financing; financial performance
**Abstract:**	Global climate change has become one of the most large-scale, widespread and far-reaching challenges facing mankind. Against this background, China has proposed a "dual-carbon" target for 2020, which greatly demonstrates China's determination and commitment to carbon emission reduction, and the heavy responsibility of realizing the "dual-carbon" target is mainly borne by heavy polluting enterprises. The burden of achieving the "dual-carbon" goal is mainly borne by heavy polluting enterprises. Although this has increased the economic burden of enterprises to a certain extent, carbon information disclosure reduces the degree of information asymmetry, and also obtains support from the government, which improves the financial performance of enterprises. Based on the data of Shanghai and Shenzhen A-share listed companies in the heavy pollution industry from 2013 to 2021, this paper analyzes the relationship between carbon information disclosure and corporate financial performance based on the information asymmetry theory, signaling theory, and rent-seeking theory. It is found that enhanced corporate carbon disclosure can significantly improve corporate financial performance, and the main effect is realized by reducing the cost of debt financing and increasing the proportion of institutional investors' shareholding, and the carbon trading policy positively moderates the relationship between the quality of carbon disclosure and corporate financial performance. In the heterogeneity analysis, this paper finds that the main effect is more significant in the samples of enterprises located in the eastern region and the central region. This study provides practical guidance for carbon disclosure and emission reduction, and provides empirical evidence for related studies .
**Order of Authors:**	Ai Ling Xu
	Yuan Yuan Su
	Yinxing Wang
**Additional Information:**	
**Question**	**Response**
**Financial Disclosure**<br><br>Enter a financial disclosure statement that describes the sources of funding for the work included in this submission. Review the submission guidelines for detailed requirements. View published research articles from *PLOS ONE* for specific examples.	Yes

This statement is required for submission and **will appear in the published article** if the submission is accepted. Please make sure it is accurate.<br><br>**Funded studies**<br>Enter a statement with the following details:<br>• Initials of the authors who received each award<br>• Grant numbers awarded to each author<br>• The full name of each funder<br>• URL of each funder website<br>• Did the sponsors or funders play any role in the study design, data collection and analysis, decision to publish, or preparation of the manuscript?<br><br>Did you receive funding for this work?	
Please add funding details.<br>as follow-up to "**Financial Disclosure**<br><br>Enter a financial disclosure statement that describes the sources of funding for the work included in this submission. Review the submission guidelines for detailed requirements. View published research articles from *PLOS ONE* for specific examples.<br><br>This statement is required for submission and **will appear in the published article** if the submission is accepted. Please make sure it is accurate.<br><br>**Funded studies**<br>Enter a statement with the following details:<br>• Initials of the authors who received each award<br>• Grant numbers awarded to each author<br>• The full name of each funder<br>• URL of each funder website<br>• Did the sponsors or funders play any role in the study design, data collection and analysis, decision to publish, or preparation of the manuscript?<br><br>Did you receive funding for this work?"	Fujian Provincial Social Science Foundation Project，grant numbers: FJ2022B082.

Powered by Editorial Manager® and ProduXion Manager® from Aries Systems Corporation

Please select the country of your main research funder (please select carefully as in some cases this is used in fee calculation).<br>as follow-up to "**Financial Disclosure**<br><br>Enter a financial disclosure statement that describes the sources of funding for the work included in this submission. Review the submission guidelines for detailed requirements. View published research articles from *PLOS ONE* for specific examples.<br><br>This statement is required for submission and **will appear in the published article** if the submission is accepted. Please make sure it is accurate.<br><br>**Funded studies**<br>Enter a statement with the following details:<br>• Initials of the authors who received each award<br>• Grant numbers awarded to each author<br>• The full name of each funder<br>• URL of each funder website<br>• Did the sponsors or funders play any role in the study design, data collection and analysis, decision to publish, or preparation of the manuscript?<br><br>Did you receive funding for this work?"	CHINA - CN
**Competing Interests**<br><br>Use the instructions below to enter a competing interest statement for this submission. On behalf of all authors, disclose any competing interests that could be perceived to bias this work—acknowledging all financial support and any other relevant financial or non-financial competing interests.<br><br>This statement is required for submission and **will appear in the published article** if the submission is accepted. Please make sure it is accurate and that any funding	The authors have declared that no competing interests exist.

sources listed in your Funding Information later in the submission form are also declared in your Financial Disclosure statement.<br><br>View published research articles from *PLOS ONE* for specific examples.<br><br>**NO authors have competing interests**<br><br>Enter: *The authors have declared that no competing interests exist.*<br><br>**Authors with competing interests**<br><br>Enter competing interest details beginning with this statement:<br><br>*I have read the journal's policy and the authors of this manuscript have the following competing interests: [insert competing interests here]*<br><br>* typeset	
**Ethics Statement**<br><br>Enter an ethics statement for this submission. This statement is required if the study involved:<br><br>• Human participants<br>• Human specimens or tissue<br>• Vertebrate animals or cephalopods<br>• Vertebrate embryos or tissues<br>• Field research<br><br>Write "N/A" if the submission does not require an ethics statement.<br><br>General guidance is provided below. Consult the submission guidelines for detailed instructions. **Make sure that all information entered here is included in the Methods section of the manuscript.**	N/A

Powered by Editorial Manager® and ProduXion Manager® from Aries Systems Corporation

**Format for specific study types**<br><br>**Human Subject Research (involving human participants and/or tissue)**<br>• Give the name of the institutional review board or ethics committee that approved the study<br>• Include the approval number and/or a statement indicating approval of this research<br>• Indicate the form of consent obtained (written/oral) or the reason that consent was not obtained (e.g. the data were analyzed anonymously)<br><br>**Animal Research (involving vertebrate animals, embryos or tissues)**<br>• Provide the name of the Institutional Animal Care and Use Committee (IACUC) or other relevant ethics board that reviewed the study protocol, and indicate whether they approved this research or granted a formal waiver of ethical approval<br>• Include an approval number if one was obtained<br>• If the study involved *non-human primates*, add *additional details* about animal welfare and steps taken to ameliorate suffering<br>• If anesthesia, euthanasia, or any kind of animal sacrifice is part of the study, include briefly which substances and/or methods were applied<br><br>**Field Research**<br><br>Include the following details if this study involves the collection of plant, animal, or other materials from a natural setting:<br>• Field permit number<br>• Name of the institution or relevant body that granted permission	
**Data Availability**<br><br>Authors are required to make all data underlying the findings described fully available, without restriction, and from the time of publication. PLOS allows rare exceptions to address legal and ethical concerns. See the PLOS Data Policy and FAQ for detailed information.	Yes - all data are fully available without restriction

Powered by Editorial Manager® and ProduXion Manager® from Aries Systems Corporation

A Data Availability Statement describing where the data can be found is required at submission. Your answers to this question constitute the Data Availability Statement and **will be published in the article**, if accepted.<br><br>**Important:** Stating 'data available on request from the author' is not sufficient. If your data are only available upon request, select 'No' for the first question and explain your exceptional situation in the text box.<br><br>Do the authors confirm that all data underlying the findings described in their manuscript are fully available without restriction?	
**Describe where the data may be found in full sentences. If you are copying our sample text, replace any instances of XXX with the appropriate details.**<br><br>• If the data are **held or will be held in a public repository**, include URLs, accession numbers or DOIs. If this information will only be available after acceptance, indicate this by ticking the box below. For example: *All XXX files are available from the XXX database (accession number(s) XXX, XXX.).*<br>• If the data are all contained **within the manuscript and/or Supporting Information files**, enter the following: *All relevant data are within the manuscript and its Supporting Information files.*<br>• If neither of these applies but you are able to provide **details of access elsewhere**, with or without limitations, please do so. For example:<br><br>*Data cannot be shared publicly because of [XXX]. Data are available from the XXX Institutional Data Access / Ethics Committee (contact via XXX) for researchers who meet the criteria for access to confidential data.*<br><br>*The data underlying the results presented in the study are available from (include the name of the third party*	All relevant data are within the manuscript and its Supporting Information files.

and contact information or URL).<br>• This text is appropriate if the data are owned by a third party and authors do not have permission to share the data.<br><br>* typeset	
Additional data availability information:	

Powered by Editorial Manager® and ProduXion Manager® from Aries Systems Corporation

Manuscript
Click here to access/download;Manuscript;Carbon information disclosure and financial performance.docx

Carbon information disclosure and corporate financial performance

—— Empirical evidence based on heavily polluting industries in China

Xu Ailing[1], Su Yuanyuan[2*], Wang Yingxin[3]

1 Business School, Huaqiao University, Quanzhou, Fujian, China

2 Business School, Huaqiao University, Quanzhou, Fujian, China

3 Business School, Huaqiao University, Quanzhou, Fujian, China

These authors contributed equally to this work.
*23013120017@hqu.stu.edu.cn

Abstract

Global climate change has become one of the most large-scale, widespread and far-reaching challenges facing mankind. Against this background, China has proposed a “dual-carbon” target for 2020, which greatly demonstrates China's determination and commitment to carbon emission reduction, and the heavy responsibility of realizing the “dual-carbon” target is mainly borne by heavy polluting enterprises. The burden of achieving the “dual-carbon” goal is mainly borne by heavy polluting enterprises. Although this has increased the economic burden of enterprises to a certain extent, carbon information disclosure reduces the degree of information asymmetry, and also obtains support from the government, which improves the financial performance of enterprises. Based on the data of Shanghai and Shenzhen A-share listed companies in the heavy pollution industry from 2013 to 2021, this paper analyzes the relationship between carbon information disclosure and corporate financial performance based on the information asymmetry theory, signaling theory, and rent-seeking theory. It is found that enhanced corporate carbon disclosure can significantly improve corporate financial performance, and the main effect is realized by reducing the cost of debt financing and increasing the proportion of institutional investors' shareholding, and the carbon trading policy positively moderates the relationship between the quality of carbon disclosure and corporate financial performance. In the heterogeneity analysis, this paper finds that the

main effect is more significant in the samples of enterprises located in the eastern region and the central region. This study provides practical guidance for carbon disclosure and emission reduction, and provides empirical evidence for related studies.

1. Introduction

Against the background of the global commitment to combating climate change and promoting carbon emission reduction, the topic of carbon information has increasingly received close attention from governments and international organizations. Consequently, corporate carbon disclosure has become a hot topic for academic discussion and an important yardstick for measuring corporate environmental responsibility and social commitment and constitutes a key link for countries around the world to actively fulfill their carbon emission reduction commitments and promote practical actions. Corporate carbon disclosure is an important way for stakeholders to understand corporate environmental dynamics and social responsibility fulfillment and provides information for investors to assess corporate value and formulate investment strategies, which promotes the greening process of the capital market. In the budding stage of carbon information disclosure, enterprises generally face the trade-off consideration between cost and benefit, and believe that information disclosure will increase the operating cost of enterprises [1], many enterprises are cautious about this, and the willingness to disclose is low, resulting in the breadth and depth of information disclosure is limited [2]. In recent years, with the spread of low-carbon concepts, as well as the community's increasing attention to environmental issues, investors' environmental preferences have increased significantly, and are more inclined to invest in enterprises that actively disclose carbon information [3]. This shift in market demand has prompted enterprises to re-examine the value of carbon disclosure, and have increased investment to improve the quality and level of disclosure [4], making carbon disclosure gradually move towards standardization and popularization. However, the degree of carbon information disclosure in China is still low, mainly due to the lack of motivation of enterprises to disclose and the lack of corresponding disclosure standards, enterprises are unwilling to spend more costs to disclose

carbon information in the form [5]. Moreover, there is a large gap in carbon information disclosure among various industries in China [6], and there are various forms of disclosure, as well as inconsistencies in the quantity and quality of disclosure [7].

With the increasingly strict national control situation on carbon emissions, carbon disclosure has become a research focus of many scholars, and studies on the relationship between carbon disclosure and corporate financial performance have become increasingly abundant. A part of scholars believe that enhanced carbon disclosure means that enterprises need to invest more resources in carbon emission reduction, which may increase the cost burden in the short term and thus negatively affect financial performance [8,9]. On the other hand, another part of scholars believe that in the long run, good carbon disclosure can enhance corporate brand image [10], strengthen market competitiveness, attract more investors and consumers who are concerned about sustainable development, and then gain more advantages in the real economy, which will ultimately improve the financial performance of enterprises [11]. Therefore, the relationship between carbon disclosure and financial performance is inconclusive and deserves further exploration. Quantitatively examining the impact of carbon disclosure on corporate financial performance and an in-depth study of its underlying mechanism is of great significance in promoting the sound development of enterprises and realizing the goal of "double carbon". Based on the data of heavy pollution listed companies in Shanghai and Shenzhen A-shares from 2013 to 2021, this paper utilizes signaling theory and rent-seeking theory to study the relationship between the quality of carbon information disclosure and the financial performance of enterprises, and finds that carbon information disclosure by enterprises can enhance the reputation of enterprises, improve the credit ratings of enterprises in financial institutions such as banks, reduce the cost of debt financing of enterprises, and then enhance the financial performance of enterprises, and carbon trading policy positively moderates the impact of carbon disclosure on the financial performance of enterprises.

The marginal contributions of this paper are: first, carbon disclosure has become an important research topic in the field of environmental accounting, and a high degree of disclosure may either bring reputation enhancement and strengthen the financial performance of firms [7]; or it may

increase the production cost of firms and reduce the efficiency of firms' outputs, which in turn may reduce financial performance [6, 12, 13]. Although more scholars have studied the relationship between carbon disclosure and financial performance, they have reached different conclusions. Based on signaling theory and rent-seeking theory, this paper explores the roles played by creditors, institutional investors, and other stakeholders in the relationship between carbon disclosure and corporate financial performance, which enriches the research perspectives and expands the existing research on the relationship between carbon disclosure and financial performance. Second, a heterogeneity test is conducted based on the regions of the sample enterprises to further analyze whether there are geographical differences in the relationship between carbon disclosure and corporate financial performance, which provides practical guidance for China's future promotion of the carbon trading market and the realization of carbon emission reduction goals.

2. Literature review

The accelerated pace of economic development has also generated more environmental pollution problems. In recent years, with the promulgation of a series of carbon-related policies in China, the research horizons of carbon disclosure in the academic community have become increasingly broad and the angles of discussion have become more and more diversified. Scholars have also conducted a lot of research on the influencing factors of carbon disclosure. From the macro level, government and investor pressure [14], the degree of legal system constraints [15], national culture [16] and so on will affect the quality of corporate carbon information disclosure. From the micro level, the political relevance of enterprises [17], the gearing ratio [18], the proportion of women in management [19], etc. will also affect the level of carbon information disclosure of enterprises to some extent. Especially with the deepening of national attention to environmental information disclosure in recent years, carbon disclosure and carbon management have gradually been linked to corporate strategy [20]. However, in terms of the economic consequences of carbon disclosure, the academic community has not reached a unified

understanding of the relationship between carbon disclosure and financial performance, but most scholars believe that there is a positive correlation between the two.

Wang et al. found that enterprises increase the degree of environmental information disclosure, can broaden the analytical perspective of analysts, and then have a positive impact on financial performance [21]. Downar with the sample of listed companies in the United Kingdom, the study shows that carbon disclosure does not significantly weaken the financial performance of the enterprise, but it may still make the enterprise produce a certain operational pressure [22]. Patrick et al. pointed out that enterprises improving the quality of carbon disclosure can effectively reduce the information asymmetry between enterprises and stakeholders, and then enhance the financial performance of enterprises [23]. Chen et al. ESG disclosure can positively affect enterprise performance, especially for enterprises with strong reputational capital and high media attention, this positive effect is more significant [24]. Further, Hahn and Uyar et al. show that firms that make carbon disclosures provide advice to key stakeholders such as shareholders and creditors to make decisions that are favorable to the firms and strengthen the supervision of environmental protection of the firms by regulators, the public, and others to optimize the environmental performance of the firms [25][26]. Li and Liu et al. find that firms' carbon disclosure quality can reduce the cost of capital, and this negative correlation is more restrictive in firms with poorer carbon performance [12]. Liesen et al. point out that specifying disclosure standards through mandatory disclosure of carbon information will enhance market efficiency and help firms to allocate more optimally in the real economy [11]. In addition, some scholars consider that carbon disclosure may have long-term effects and propose to examine the effectiveness of carbon disclosure from two perspectives, i.e., examining the current cash flow status of enterprises in the short term, and making a good valuation of future profitability in the long term [27].

However, some scholars believe that with the deepening of carbon disclosure, the financial performance of enterprises may suffer negative impacts and deterioration. The legitimacy theory requires enterprises to satisfy the implicit social contract of stakeholders to maintain their survival and development [28]. Therefore, when enterprises face more serious pollution problems, to maintain their legitimacy status, they will be forced to conduct information disclosure [29] to divert investors' attention from their behavior [30], and the effectiveness of information disclosure

is difficult to guarantee. When enterprises face government pressure to make carbon disclosure, enterprises with poor environmental performance may make involuntary and low-credibility disclosures, or may even make false disclosures, to salvage their image. Empirical studies have shown that firms with a high frequency of voluntary disclosure may have surplus manipulation before equity refinancing, and share prices and earnings will drop significantly after financing [31], which provides evidence of the economic consequences of voluntary disclosure by firms. In addition, the costs invested by firms to take on environmental responsibility will lead to the diversion of resources, affecting the improvement of firms' financial performance. Siddique et al. points out that in the short term, enhanced carbon disclosure by firms may lead to a negative trend due to a temporary decline in financial performance as a result of initial investment costs, management adjustments, and other factors. However, in the long run, carbon disclosure can enhance corporate image, increase investor confidence, promote effective allocation of resources, and ultimately have a positive impact on financial performance [10]. Plumlee et al. found through an empirical study that, in the short run, carbon disclosure will be accompanied by additional cost inputs, regulatory pressures, and uncertainty of market responses, and this behavior will instead lead to a corporate financial performance decline [9]. Zhang Jing et al. found that the improvement of the quality of carbon disclosure will reduce the financial performance of the enterprise, but the carbon performance of the enterprise shows a significant positive correlation with the financial performance [32].

By analyzing the existing literature, it can be found that with the rapid development of the economy and the increasing prominence of environmental pollution, carbon disclosure has become the focus of academic attention. The research perspectives are gradually diversified, covering multiple levels such as government policy, legal system, corporate culture, and internal corporate governance structure. Although most scholars agree that carbon disclosure is positively related to financial performance, and emphasize its positive effects on corporate image, market trust, and resource allocation, there are still differences in academic perceptions of this relationship. Some studies point out that in the short term, carbon disclosure may put pressure on corporate finances due to increased costs and market uncertainty, i.e., there is a negative correlation between carbon disclosure and financial performance.

3. Theory and hypothesis

Scholars who hold the signaling theory believe that carbon disclosure can effectively enhance firm value[33]. The reason is that high-quality disclosure can reduce the information asymmetry between enterprises and stakeholders, and enhance stakeholders' trust in enterprises. As an informal system, the deepening of social trust reduces the friction in the transaction process between the two parties[34], enabling enterprises to reach cooperation at a lower cost, which in turn helps them to obtain bank loans and equity financing [35], and ultimately leads to an increase in the financial performance of firms that make carbon disclosures[36]. On the contrary, if firms do not make disclosures, the degree of information asymmetry between firms and stakeholders is exacerbated. As it is difficult for investors to obtain data on the environmental activities carried out by firms, it is more difficult to assess the risks and benefits arising from the firms' activities. As a result, investors need to spend a large cost to search for information on the environmental activities of enterprises, and this cost will eventually be borne by enterprises that do not disclose information[37], which ultimately affects the financial performance of enterprises.

Also supporting this view is the rent-seeking theory, which explains the positive relationship between the quality of carbon disclosure and financial performance from the perspective of social responsibility. Rent-seeking activity refers to the process in which enterprises utilize their association with the government to obtain excess "rent" income in the process of government control and intervention in market economic activities [38]. Through active rent-seeking activities, firms can effectively establish a strong government-enterprise relationship, and then obtain more financial subsidies and key resource support from the government [39]. In addition, these activities can, to a certain extent, mitigate the negative impact of market failure on business operations and ensure that the stability of corporate finance is effectively maintained[40]. It has been found that corporate social responsibility is a kind of rent-seeking behavior [41]. By fulfilling social responsibility, enterprises form a good reputation in front of the public, which can not only help enterprises to reduce the cost of debt financing[42], show the government that the enterprise has contributed to society, and form an implicit exchange of benefits with the government, to obtain

more resources to support [39]. Comparatively, the resources allocated by local governments will be tilted to enterprises with a higher degree of social responsibility fulfillment [41], and the external disclosure of environmental information is precisely the social responsibility that enterprises must fulfill. Therefore, carbon disclosure by enterprises can be regarded as enterprises seeking rent from the government to seek some benefits on the economic level, such as giving enterprises appropriate tax breaks and corresponding financial subsidies [41], and the tilting of these financial resources enhances the financial performance of enterprises.

In summary, from the perspective of signaling theory and rent-seeking theory, the disclosure of carbon information by enterprises can reduce the degree of information asymmetry between enterprises and stakeholders [43], enhance the trust of the outside world in the enterprise, access to better quality resources, and thus enhance the financial performance of enterprises; enterprises for rent-seeking, for the local government to take on a certain degree of environmental protection responsibility, can obtain the policy tilt to enhance the financial performance of enterprises; enterprises for rent-seeking, for the local government to take on certain environmental protection responsibility, can obtain the policy tilt to enhance the financial performance of enterprises. The financial performance of enterprises can be enhanced by obtaining policy favoritism. Based on this, this paper puts forward the following hypotheses :

H1: Other things being equal, there is a positive contribution of firms' carbon disclosure to firms' financial performance.

Give the significant / No.

4. Methodology

4.1 Sample selection and data source

This paper selects the heavy pollution enterprises listed in Shanghai and Shenzhen A-shares from 2013 to 2021. The data in this paper are selected from 2013 because since the “Five-in-one” was proposed in the report of the “18th National Congress” in 2012, enterprises gradually began to pay attention to the disclosure of environmental information, and the environmental protection data at this time are easier to obtain and have reference significance. For the selection of heavily polluting enterprises, reference to the study of Pan Ailing to determine [44]. According to this

criterion, a total of 5984 observations from 1016 listed companies are screened out in this paper, of which the sample with disclosed carbon information is 1836 observations, and after excluding the samples with missing certain indexes, excluding ST samples, samples of financial enterprises, and samples with missing data, and shrinking the upper and lower 1% tail of the main variables, the remaining 299 listed companies have 1612 observations. Corporate financial data from Cathay Pacific (CSMAR) database.

4.2 Measurement model

To test hypotheses 1 , the model is designed as follows:

$$Roe_{i,t}=\beta_0+\beta_1Cdi_{i,t}+\beta_2Age_{i,t}+\beta_3Invent_{i,t}+\beta_4Bsize_i+\beta_5Jsize_{i,t}+\beta_6Lev_{i,t}+\beta_7Growth_{i,t}+\beta_8Intang_{i,t}+\sum Industry+\sum Year+\varepsilon_{i,t} \quad (1)$$

where Roe $_{i,t}$ is the financial performance of firm i in year t and $Cdi_{i,t}$ is the carbon disclosure of firm i in year t. In the model, β_0 is a constant term, β_i is the regression coefficient of each variable, ε is a random perturbation term, and year and industry fixed effects are controlled. If the empirical results show that β_1 is positive, then H1 is proved, i.e., the carbon disclosure positively affects corporate financial performance.

4.3 Variable definition

4.3.1 Dependent variable

In this paper, Roe (return on net assets) is selected as the core proxy variable. Roe, as a key indicator of return on shareholders' equity, can intuitively reflect the efficiency of enterprises in utilizing shareholders' funds to create profits. In contrast, although Roe (return on total assets) is also an important indicator of corporate profitability, Roe evaluates the profitability of the overall assets from the perspective of the total assets of the enterprise, and the size and composition of the total assets of different industries tend to differ greatly due to the differences in the capital structure, business strategies, and market environment, which may result in Roe in a direct

comparison of the profitability of enterprises in different industries. This makes Roe's direct comparison of the profitability of companies in different industries may have certain limitations. By focusing on shareholders' equity, Roe excludes the differences in total asset size due to industry characteristics, and thus can more accurately reveal the performance of enterprises in utilizing their capital for value creation, as well as the economic impacts on long-term operating results due to the implementation of strategic initiatives such as carbon disclosure. Therefore, this paper chooses Roe as a proxy variable for financial performance.

4.3.2 Independent variable

Referring to Li Li's (2019) study [16], this paper constructs a system for evaluating corporate carbon disclosure. Using Cdi as a proxy variable for measuring corporate carbon disclosure, a total of four aspects are evaluated carbon emission reduction strategy and management, carbon emission reduction accounting, carbon emission reduction measures, and carbon emission reduction performance, and the specific scoring items are shown in Table 1. Final application of the entropy method to construct a composite indicator for carbon disclosure.

Table 1. Carbon Disclosure Indicators

**Level 1 indicators**	**Secondary indicators**	**explain**
Carbon emission reduction strategy and management	Carbon emission reduction institutions setting up	An organization is set up to get 1 point, otherwise 0 points
	Strategy and goal	1 for qualitative description, 2 for quantitative description, otherwise 0
	Investment in energy conservation projects	
	The implementation of the national emission reduction policies	
	Enterprise internal environmental protection assessment system	

	Solid waste discharge volume	
Carbon emission reduction accounting	Accounting for greenhouse gas emissions	
	Accounting for greenhouse gas emissions	
	Accounting for the emission intensity of major pollutants	
	Report the main distribution of emissions	
Carbon emission reduction measures	Purchase and upgrade environmental protection facilities	
	Develop low-carbon technology and low-carbon products	
	Research and development of pollution prevention technology and process	
	Pollution monitoring equipment	
Carbon emission reduction performance	Whether the subject responsibility is clear	
	Emission-reduction performance appraisal method	
	Financial subsidies for carbon emission reduction	1 point for the subsidy that year, otherwise 0 points

4.3.3 Control variables

We control the correlation variables concerning the studies of Wen Subin [45], Shen Hongtao [46], and Song Xiaohua [47]. The relevant variables are shown in the following table.

Table 2. Description of variables

**type of variable**	**Variable name**	**variable symbol**	**Variable measure**
explained variable	financial performance	Roe	Net income after tax to average shareholders' equity
explanatory variable	carbon information disclosure	Cdi	The index construction method is shown above
controlled variable	enterprise age	Age	Fiscal year-establishment year + 1
	Inventory intensity	Invent	The ratio of the Inventory balance to the total assets at the end of the period
	Intangible asset density	Intang	The ratio of the net Intangible assets to the total assets at the end of the period
	asset-liability ratio	Lev	The ratio of ending total liabilities to total assets
	Board size	Bsize	Natural logarithm of the board count
	The board of supervisors size	Jsize	Natural logarithm of the number of supervisory boards
	Enterprise growth ability	Growth	increase rate of business revenue
	a particular year	Year	Virtual variables representing the year

	trade	Industry	Virtual variables representing the industry

5. Empirical analysis

5.1 Descriptive statistics

According to Table 3, the maximum value of financial performance (Roe) is 0.363, the minimum value is -0.809, the difference is 1.172, and the mean value is 0.062, which indicates that there is a big difference in profitability among Chinese heavy pollution listed enterprises and the overall input-output level of the industry is low. The mean and median of carbon disclosure (Cdi) are 0.085 and 0.062 respectively, indicating that the disclosure of heavy pollution listed enterprises is low; the minimum and maximum values are 0.004 and 0.328 respectively, indicating that the quality of disclosure is mixed and there are large differences among different enterprises. In addition, the rest of the variables are in the reasonable range.

Table 3. Descriptive statistics

Variable	N	Mean	SD	Min	p25	p50	p75	Max
Roe	1612	0.062	0.135	-0.809	0.028	0.065	0.123	0.363
Cdi	1612	0.085	0.072	0.004	0.029	0.062	0.119	0.328
Liability	1612	0.027	0.013	0.000	0.018	0.027	0.035	0.060
INVH	1612	0.553	0.224	0.004	0.409	0.582	0.714	0.929
Age	1612	20.67	5.007	8.000	17.000	21.000	24.000	34.000
Bsize	1612	2.401	0.261	1.609	2.197	2.398	2.565	2.944
Jsize	1612	1.542	0.383	1.099	1.099	1.609	1.792	2.398
Growth	1612	0.149	0.330	-0.456	-0.030	0.0850	0.244	2.108
Intang	1612	0.055	0.052	0.001	0.023	0.041	0.066	0.326
Invent	1612	0.105	0.089	0.001	0.039	0.085	0.142	0.408
Lev	1612	0.499	0.180	0.059	0.371	0.510	0.639	0.908

5.2 Correlation analysis

By calculating the Pearson correlation coefficient, Table 4 can be obtained. According to the table, the correlation coefficient between Roe and Cdi is 0.065, which is significant at the 1%

level, which indicates that corporate carbon disclosure has a significant positive impact on financial performance in the whole sample, and this finding is the same as the existing literature [21] [23] [24]. In addition, the absolute value of the correlation coefficients of the variables is less than 0.5, which indicates that there is no serious multicollinearity relationship among the variables. For the variables whose absolute values of the correlation coefficients in the table are between 0.3 and 0.6, this paper further conducts the VIF test. The results show that the VIF values of all independent variables are less than 5, which means that there is no multicollinearity problem.

Table 4. Correlation analysis

lity	INVH	Age	Bsize	Jsize	Growth	In
***	1					
10	0.00300	1				
***	0.238***	0.087***	1			

5.3 Main effect regression

To test the relationship between carbon disclosure and corporate financial performance, this paper takes the Roe of the enterprise in the current period as the explanatory variable and the carbon disclosure of the enterprise as the explanatory variable, and the results are shown in Table 5. In Table 5, industry and year fixed effects are controlled respectively, and the results are all significant, and this regression result verifies H1, i.e., carbon disclosure by enterprises can enhance corporate financial performance. Taking column (3) as an example, when Cdi increases by one standard deviation, Roe improves by about 19.85%（0.072*0.171/0.062）, which is significant in terms of economic effect, and H1 is verified.

Table 5. Regression results of carbon disclosure and corporate financial performance

	**Roe**		
	**(1)**	**(2)**	**(3)**
Cdi	0.189***	0.199***	0.171***
	(0.037)	(0.038)	(0.037)
Age	0.001**	0.001	-0.001
	(0.001)	(0.001)	(0.001)
Bsize	-0.009	-0.012	-0.009
	(0.014)	(0.014)	(0.014)
Jsize	0.006	0.006	0.008
	(0.009)	(0.009)	(0.009)
Growth	0.108***	0.107***	0.099***
	(0.012)	(0.012)	(0.012)
Intang	-0.103**	0.021	0.037
	(0.045)	(0.062)	(0.060)
Invent	-0.059*	-0.062	-0.063
	(0.034)	(0.043)	(0.042)
Lev	-0.215***	-0.221***	-0.208***
	(0.028)	(0.029)	(0.029)
Industry effect	NO	YES	YES
Year effect	NO	NO	YES
constant term	0.136***	0.117***	0.130***
	(0.033)	(0.036)	(0.035)
Number of observed samples	1612	1612	1612
R^2	0.158	0.161	0.179

Note: *, **, and *** are significant at 10%, 5%, and 1% levels, respectively.

5.4 Robustness test

5.4.1 Instrumental variable approach

Since there may be an endogenous causality between carbon disclosure quality and firms' financial performance, i.e., growth in firms' financial performance may motivate firms to improve their carbon disclosure quality. To alleviate this problem as well as the problem of omitted variables, this paper uses the instrumental variable method for regression analysis. For the selection of instrumental variables, this paper takes the average value of carbon disclosure in the year of the industry to which the sample enterprise belongs (ACdi) and the lagged carbon disclosure of the enterprise (LCdi) as instrumental variables. If the quality of carbon disclosure in the year of the industry to which the enterprise belongs is high, it will drive the enterprise to improve the quality of carbon disclosure in the year, while the impact on the enterprise's financial performance is relatively small; the carbon disclosure of the lagged carbon disclosure of the enterprise in the period of one period Similarly, the carbon disclosure of two consecutive periods has a higher correlation, while the carbon disclosure of one lagging period has a smaller impact on the financial performance of the enterprise in the current period. Therefore, the selection of instrumental variables meets the requirements. The regression results are shown in Table 6. （ Columns (1) and (2) show the regression results of taking the average value of carbon disclosure in the year (ACdi) of the industry to which the sample firms belong as an instrumental variable. The first-stage regression results show that ACdi is positively correlated with the explanatory variable Cdi at the 1% level, as expected. Column (2) shows the results of the second-stage regression, which is significantly positive at the 10% level, and the Cragg-Donald Wald F-value is 275.41, which rejects the hypothesis of “weak instrumental variables”.The Kleibergen-Paap rk LM-value is 135.12, which is at the 1% level, and the Kleibergen-Paap rk LM-value is 135.12, which is at the 1% level. The Kleibergen-Paap rk LM value is 135.12, which is significant at the 1% level. Columns (3) and (4) show the results of regressing firms' one-period lagged carbon disclosure (LCdi) as an instrumental variable. The first-stage regression results show that LCdi is

positively correlated with the explanatory variable Cdi at the 1% level, as expected. Column (2) shows the results of the second-stage regression, which is significantly positive at the 1% level and the Cragg-Donald Wald F-value is 1723.214, which rejects the hypothesis of "weak instrumental variable".The Kleibergen-Paap rk LM-value is 271.93, which is at the 1% level. The Kleibergen-Paap rk LM value is 271.93 which is significant at 1%. Therefore, after correcting for endogeneity problem, the conclusion of this paper is still valid.

Table 6. Instrumental variables regression results

	（1）	（2）	（3）	（4）
	Cdi	***Roe***	***Cdi***	***Roe***
	**stage Ⅰ**	**stage Ⅱ**	**stage Ⅰ**	**stage Ⅱ**
ACdi	0.906***			
	(0.055)			
LCdi			0.773***	
			(34.37)	
Cdi		0.203*		0.168***
		(0.114)		（3.06）
Age	-0.001***	0.000	-0.001*	0.001
	(0.000)	(0.00)	(-1.76)	(0.68)
Bsize	0.014*	-0.010	0.003	-0.000
	(0.007)	(0.014)	(0.61)	(-0.03)
Jsize	0.006	0.008	-0.005	0.008
	(0.005)	(0.010)	(-1.26)	(0.72)
Growth	0.003	0.099***	0.003	0.126***
	(0.005)	(0.010)	(0.65)	(7.89)
Intang	-0.035	0.037	0.014	0.029
	(0.039)	(0.073)	(0.47)	(0.42)
Invent	-0.005	-0.063	0.013	-0.015
	(0.021)	(0.041)	(0.82)	(-0.34)
Lev	0.033***	-0.209***	0.011	-0.229***
	(0.010)	(0.019)	(1.39)	(-6.69)
Industry effect	YES	YES	YES	YES
Year effect	YES	YES	YES	YES
constant term	-0.021	0.189***	0.031**	0.141***
	(0.020)	(0.037)	(2.11)	(3.27)
Number of observed samples	1612	1612	1258	1258

R^2	0.216	0.189	0.615	0.207
Phase I F-value	275.41		1181.09	
Kleibergen-Paap rk LM value	135.12***		271.93***	
Cragg-Donald Wald F value	274.462		1723.214	

Note: *, * * , and * * * are significant at 10%, 5% , and 1% levels, respectively.

5.4.2 Explanatory variables lagged two period

Some studies suggest that larger or better-performing firms have stronger incentives to make disclosures [48][49][50], i.e., there may be a reverse causation problem between the two. To solve the possible reverse causality problem in the subtext, this paper uses the carbon disclosure lagged by two periods instead of the current period to conduct the regression, and the results, as shown in column (1) of Table 7, are still significantly positively correlated, so the conclusion of this paper is robust.

5.4.3 Shorter sample periods

To eliminate the impact of the new crown epidemic on firms' operations, this paper excludes data from 2020-2021 to ensure the robustness of the regression results. The results, as shown in column (2) of Table 7, show that the relationship between carbon disclosure quality and firms' financial performance remains significantly positive. Therefore, excluding the effect of the new crown epidemic, the improvement of carbon disclosure quality can still promote the growth of corporate financial performance.

5.4.4 Substitution of explanatory variables

To ensure that the results are robust, this paper uses Roa as a proxy variable for Roe in the regression, and the regression results, as shown in column (3) of Table 7, show that Cdi is still positively correlated with Roa, indicating that the results are robust.

Table 7. Robustness test results

	（1）	（2）	（3）
	Roe	***Roe***	***Roa***
L2Cdi	0.151***		
	(0.043)		

Cdi		0.176***	0.069***
		（0.045）	（0.014）
Age	0.000	-0.001	-0.000
	(0.001)	(0.001)	(0.000)
Bsize	0.010	-0.002	-0.003
	(0.017)	(0.017)	(0.005)
Jsize	0.007	0.007	0.001
	(0.012)	(0.012)	(0.003)
Growth	0.119***	0.097***	0.038***
	(0.017)	(0.014)	(0.005)
Intang	0.042	0.073	0.019
	(0.079)	(0.073)	(0.024)
Invent	-0.034	-0.041	-0.054***
	(0.051)	(0.052)	(0.014)
Lev	-0.200***	-0.247***	-0.118***
	(0.036)	(0.035)	(0.008)
Industry effect	YES	YES	YES
Year effect	YES	YES	YES
constant term	0.011	0.125***	0.084***
	(0.047)	(0.042)	(0.013)
Number of observed samples	1019	1166	1612
R^2	0.210	0.200	0.282

Note: *, * * , and * * * are significant at 10%, 5% , and 1% levels, respectively.

5.5 Analysis of mediating role

5.5.1 The intermediary role of institutional investors' shareholdings

Under the trend that all parties in society pay more and more attention to environmental protection, the capital market also pays more attention to the fulfillment of corporate social responsibility, and investors will have a greater possibility to invest in enterprises with a high degree of social responsibility fulfillment. Carbon disclosure, as an important part of corporate social responsibility fulfillment, is also a concern for various types of investors [51]. Compared with individual investors, institutional investors have more specialized knowledge reserves,

richer information sources, and a more comprehensive interpretation of the national green economy development trend, and therefore are more likely to notice the enterprises' investment in social responsibility and information disclosure [52] and thus make investment decisions.

From the perspective of principal-agent theory, first of all, institutional investors, as external stakeholders of enterprises, are affected by the quality of corporate disclosure in their investment decisions. In the principal-agent relationship, due to information asymmetry and conflict of interest, the agent may take behaviors that are inconsistent with the interests of the principal, which increases the agency cost. When companies disclose high-quality carbon information, institutional investors, as external stakeholders of companies, can better understand the environmental management status and potential risks of companies, which reduces the degree of information asymmetry. This not only helps institutional investors to more accurately assess the value and risk of the company and reduce the agency costs arising from insufficient information but also increases their investment confidence in the enterprise, which in turn leads to more informed investment decisions and higher shareholding ratios. Second, institutional investors, as external shareholders, have a stronger incentive and ability to obtain internal information about the enterprise, monitor the decisions and behaviors of corporate executives, inhibit inefficient investment [53], and prompt the enterprise to act by the principle of maximizing shareholders' interests. This helps to reduce moral hazard and conflict of interest within the enterprise, improve the efficiency of corporate governance, and then increase the financial performance of the enterprise. Finally, improving the quality of carbon information disclosure can enhance the reputation of the enterprise, which helps attract more institutional investors to pay attention to and invest in the enterprise. Therefore, improving the quality of carbon disclosure is not only conducive to the transparency of its environmental management but also an important means of attracting institutional investors and optimizing the investment structure.

5.5.2 Intermediation of debt financing costs

Principal-agent theory points out that, in the environment of a market economy, there exists the phenomenon of information asymmetry between the enterprise and the stakeholders, due to the

separation of ownership and operation rights, the enterprise operator tends to grasp more internal information, making it difficult for stakeholders to have a comprehensive insight into the business reality of the enterprise, which is easy to induce the risk of adverse selection. At the same time, as an agent of the enterprise operators, in the process of pursuing their interests, they may ignore or harm the rights and interests of stakeholders, which in turn affects the effective allocation of market resources and increases the cost of enterprise financing. However, the improvement of the quality of corporate disclosure can reduce the degree of information asymmetry between enterprises and stakeholders [54], help stakeholders understand the risks and performance of enterprises, and thus make it easier for enterprises to obtain financing [55].

First, from the principal-agent theory, agency costs include monitoring costs, incentive costs, and residual losses. When enterprises improve the quality of carbon disclosure, it becomes easier for creditors to supervise the environmental management of enterprises, thus reducing the supervision cost. At the same time, creditors can also more accurately assess the environmental risks and sustainability of the enterprise, thus designing incentive contracts more rationally and reducing incentive costs. All these help to reduce the agency cost of enterprises and thus the cost of debt financing. Second, the quality of corporate disclosure is likewise an important aspect of its fulfillment of social responsibility. By fulfilling social responsibility, the reputation and corporate value of enterprises are enhanced, stabilizing the psychological expectations of both investment and financing parties, reducing friction in the transaction process, and lowering the costs required to reach cooperation [56]. For example, banks' trust in firms can help firms obtain lower loan interest rates [57]. As a result, firms that have a high reputation through disclosure have relatively low debt financing costs. Finally, corporate disclosure of carbon information is not only a concrete manifestation of its social responsibility but also a strategy to seek resource support from the government. Through the disclosure of social responsibility information, enterprises can establish closer ties with the government and all walks of life, and obtain more policy and resource support, which can help them break through development bottlenecks and obtain more convenient financing opportunities [58]. At the same time, the social responsibility performance of enterprises also affects the audit results of the regulatory authorities, which in turn affects their financing costs [57].

Based on the above analysis, firms that improve the quality of carbon disclosure have the potential to both reduce the cost of debt financing they face and increase their institutional investor shareholding. To verify this relationship, this paper designs the following model:

$$Roe_{i,t}=\beta_0+\beta_1 CDI_{i,t}+\beta_2 Age_{i,t}+\beta_3 INVENT_{i,t}+\beta_4 Bsize_{i,t}+\beta_5 Jsize_{i,t}+\beta_6 Lev_{i,t}+\beta_7 Growth_{i,t}+\beta_8 INTANG_{i,t}+\Sigma Industry+\Sigma Year+\varepsilon_{i,t} \quad (2)$$

$$Mediate_{i,t}=\alpha_0+\alpha_1 CDI_{i,t}+\alpha_2 Age_{i,t}+\alpha_3 INVENT_{i,t}+\alpha_4 Bsize_{i,t}+\alpha_5 Jsize_{i,t}+\alpha_6 Lev_{i,t}+\alpha_7 Growth_{i,t}+\alpha_8 INTANG_{i,t}+\Sigma Industry+\Sigma Year+\varepsilon_{i,t} \quad (3)$$

$$Roe_{i,t}=\gamma_0+\gamma_1 CDI_{i,t}+\gamma_2 Mediate_{i,t}+\gamma_3 Age_{i,t}+\gamma_4 INVENT_{i,t}+\gamma_5 Bsize_{i,t}+\gamma_6 Jsize_{i,t}+\gamma_7 Lev_{i,t}+\gamma_8 Growth_{i,t}+\gamma_9 INTANG_{i,t}+\Sigma Industry+\Sigma Year+\varepsilon_{i,t} \quad (4)$$

Among them, model (2) is the same as the model (1), and $Mediate_{i,t}$ in the model (3) is the mediating variable. To verify the mechanism of carbon disclosure quality on corporate financial performance, this paper selects two indicators of corporate debt financing cost (Liability) and institutional investor shareholding ratio (INVH) as mediating variables. Among them, Liability refers to the practice of Li Guangzi, which is measured by the ratio of the amount of the interest expense detail account in the finance cost to the average total liabilities of the enterprise [59]; $INVH_{i,t}$ is the proportion of institutional investors' shareholding of firm i in year t. The regression results are shown in Table 8. The regression results are shown in Table 8. In the analysis of the mediating role of debt financing cost, Liability is significantly negatively related to *Cdi*; after *Liability* is added to the regression, *Cdi* is significantly positively related to Roe, and Liability is significantly negatively related to *Roe*. In the analysis of the mediating role of institutional investors' shareholding, INVH is significantly positively related to *Cdi*; after *INVH* is added to the regression, *Cdi* is significantly positively related to *Roe*, and *INVH* is significantly negatively related to *Roe*. This indicates that the cost of debt financing and institutional investors' shareholding play a mediating role between carbon disclosure quality and corporate financial performance.

Table 8. Analysis of the role of intermediaries

	Institutional investor holdings			Cost of debt financing		
	Roe	***INVH***	***Roe***	***Roe***	***Liability***	***Roe***
Cdi	0.171***	0.508***	0.132***	0.171***	-0.005	0.163***
	(0.037)	(0.062)	(0.036)	(0.037)	(0.003)	(0.037)
INVH			0.075***			
			(0.016)			
Liability						-1.459***
						(0.314)
Age	-0.001	-0.002**	-0.000	-0.001	0.000	-0.000
	(0.001)	(0.001)	(0.001)	(0.001)	(0.000)	(0.001)
Bsize	-0.009	0.021	-0.011	-0.009	0.001	-0.008
	(0.014)	(0.022)	(0.014)	(0.014)	(0.001)	(0.014)
Jsize	0.008	0.118***	-0.001	0.008	-0.001	0.007
	(0.009)	(0.015)	(0.009)	(0.009)	(0.001)	(0.009)
Growth	0.099***	0.012	0.098***	0.099***	0.002***	0.103***
	(0.012)	(0.017)	(0.013)	(0.012)	(0.001)	(0.012)
Intang	0.037	-0.227**	0.054	0.037	0.007	0.047
	(0.060)	(0.103)	(0.060)	(0.060)	(0.006)	(0.059)
Invent	-0.063	-0.265***	-0.043	-0.063	-0.003	-0.067
	(0.042)	(0.060)	(0.043)	(0.042)	(0.004)	(0.042)
Lev	-0.208***	0.197***	-0.222***	-0.208***	0.031***	-0.163***
	(0.029)	(0.030)	(0.030)	(0.029)	(0.002)	(0.029)
Industry effect	YES	YES	YES	YES	YES	YES
Year effect	YES	YES	YES	YES	YES	YES
constant term	0.130***	0.360***	0.103***	0.130***	0.013***	0.150***
	(0.035)	(0.055)	(0.035)	(0.035)	(0.003)	(0.035)
Number of observed samples	1612	1612	1612	1612	1612	1612
R^2	0.189	0.278	0.200	0.189	0.359	0.201

Note: *, **, and *** are significant at the 10%, 5% and 1% levels, respectively

5.6 Further analysis

To further refine the study of the relationship between carbon disclosure and financial performance, this paper takes the region where the sample enterprises are registered as the criterion and divides all the enterprises into eastern, central, and western regions for group regression, and the regression results are shown in Table 9. The positive correlation between

carbon disclosure and financial performance is significant in the samples of enterprises in the eastern region and the western region, while it is not significant in the samples of enterprises in the western region, and there may be several reasons for this difference. First, as the level of regional economic development increases, investors will pay more attention to the environmental information of enterprises and take it as one of the considerations for investment decisions. Enterprise disclosure of environmental information can better meet the needs of investors, reduce the cost of equity capital of enterprises [60], and enhance financial performance. The degree of economic development in the eastern and central regions is higher than that in the western region, so investors in the central and eastern regions pay more attention to the fulfillment of corporate environmental responsibility, and the positive correlation between carbon information disclosure and financial performance is more significant. Secondly, regions with a higher degree of economic development tend to have a more complete environmental information disclosure mechanism. This can not only improve market transparency and reduce information asymmetry [23] but also form an effective external supervision of enterprises [61], prompting enterprises to pay more attention to environmental protection and the quality of carbon information disclosure, thus further enhancing the positive relationship between carbon information disclosure and financial performance. Finally, enterprises in the central and eastern regions are more active in innovation investment and promote green development and carbon emission reduction through technological innovation [62]. The higher level of technology makes enterprises in the central and eastern regions more advantageous in green development and carbon emission reduction, which helps to improve the environmental image and market competitiveness of enterprises, and thus promotes the improvement of corporate financial performance.

Table 9. Heterogeneity test

	Roe		
	**Eastern Region**	**Central Region**	**Western region**
Cdi	0.139***	0.345***	0.193
	(0.046)	(0.112)	(0.136)
Age	0.000	-0.004*	-0.002
	(0.001)	(0.002)	(0.002)

Bsize	0.008	-0.049	-0.068
	(0.014)	(0.033)	(0.050)
Jsize	-0.007	0.110***	-0.002
	(0.010)	(0.026)	(0.035)
Growth	0.090***	0.070***	0.155***
	(0.010)	(0.023)	(0.030)
Intang	-0.038	0.493***	-0.067
	(0.081)	(0.190)	(0.183)
Invent	-0.065	-0.194*	-0.130
	(0.044)	(0.104)	(0.140)
Lev	-0.104***	-0.357***	-0.255***
	(0.021)	(0.046)	(0.052)
Industry effect	YES	YES	YES
Year effect	YES	YES	YES
constant term	0.072*	0.157*	0.265**
	(0.037)	(0.084)	(0.114)
Number of observed samples	964	368	280
R^2	0.143	0.308	0.323

Note: *, **, and *** are significant at 10%, 5%, and 1% levels, respectively.

6. Conclusions and policy implications

In order to vigorously promote the realization of the "double carbon" goal, the State implements strict management of carbon emissions by enterprises, and at the same time introduces various policies to improve the level and quality of carbon information disclosure. However, is carbon disclosure contrary to the goal of corporate profitability, i.e., does corporate carbon disclosure behavior affect corporate financial performance? Based on this question, this paper investigates the impact of carbon disclosure on financial performance using a sample of listed companies in heavy pollution industries listed on Shanghai and Shenzhen A-shares in China from 2013-2021, and analyzes its mediating role in order to study the relationship between the two. It is found that the improvement of the quality of corporate carbon disclosure can enhance the financial performance of enterprises, and this result is still significant after considering the endogeneity problem and conducting robustness tests. This paper further investigates the mechanism by which the quality of carbon disclosure positively affects the financial performance of enterprises and finds that the improvement of carbon disclosure quality can reduce the cost of debt financing and

increase the proportion of institutional investors' shareholding, which in turn improves the financial performance of enterprises. In addition, the results of the heterogeneity test show that the main effect is more significant in the sample of firms located in the eastern region versus the central region.

Based on the above research, this paper suggests the following points to enterprises:

First, enterprises must deeply understand and attach great importance to the importance of carbon information disclosure, and actively take action to not only increase the disclosure of carbon information in terms of quantity but also improve the breadth and depth of the disclosure in terms of quality, so that all sectors of the society can fully and accurately understand the carbon emissions of enterprises. While pursuing economic benefits, enterprises should not neglect their responsibility and commitment to the environment. Enterprises should take into account short-term benefits and long-term development, and carry out the concept of low-carbon and environmental protection in every aspect of production and operation. Through technological innovation, management optimization, and other means, they can reduce the carbon emission intensity of enterprises and enhance the efficiency of resource utilization. Carbon disclosure is not only a channel for enterprises to transmit information to the outside world but also a bridge for enterprises to communicate with stakeholders. Enterprises should ensure the accuracy and transparency of carbon information disclosure so that stakeholders can clearly understand the enterprise's carbon emissions and make more informed decisions. At the same time, high-quality carbon disclosure can attract more institutional investors who are looking for long-term and stable returns, thus helping enterprises achieve solid and significant growth in financial performance.

Second, companies should abandon the traditional concept of carbon disclosure as an integral part of their overall development strategy rather than as an additional burden. With society's growing concern for environmental protection and sustainable development, carbon disclosure has become an important indicator of a company's green image and market competitiveness. Previous studies have confirmed that by improving the quality of carbon disclosure, enterprises can effectively reduce their debt financing costs and increase the proportion of institutional investors' shareholding, which suggests that the enhanced quality of carbon disclosure can provide an intrinsic driving force for the growth of an enterprise's financial performance. Therefore,

enterprises should regard carbon disclosure as an important means to promote sustainable development, enhance the initiative and enthusiasm for information disclosure, and ensure that the disclosed information can truly and objectively reflect the carbon emissions of enterprises.

Thirdly, enterprises located in the eastern region should make full use of their geographical advantages to demonstrate their environmental responsibility and green business achievements, which not only helps attract more investors and partners, but also enhances their market competitiveness. To further enhance green competitiveness, enterprises should focus on technological innovation to reduce carbon emissions and improve resource utilization efficiency. At the same time, enterprises should pay close attention to the dynamics of green policies, adjust their business strategies in a timely manner according to policy changes, and seize the opportunities brought by the policies.

Fourth, enterprises should grasp the development opportunities brought by national policy support. At present, the state has issued relevant laws and regulations to regulate corporate information disclosure behavior, enterprises should strictly abide by the relevant system, actively fulfill their social responsibility, proactively disclose carbon information, and contribute to the realization of the “double carbon” goal. By improving the quality of carbon disclosure, enterprises can not only win the recognition and support of creditors and other stakeholders but also lay a solid foundation for their long-term development. In the increasingly fierce market competition, only those enterprises that are truly concerned about environmental protection and actively fulfill their social responsibilities can stand out from the market and achieve sustainable development.

Author Contributions

Conceptualization: Ailing Xu, Yuanyuan Su.

Data curation: Yuanyuan Su, Yinxing Wang.

Methodology: Ailing Xu, Yuanyuan Su, Yinxing Wang.

Writing – original draft: Ailing Xu, Yuanyuan Su.

Writing – review & editing: Ailing Xu, Yuanyuan Su.

Funding: This research was supported by the Fujian Provincial Social Science Foundation Project, grant numbers: FJ2022B082.

Data Availability Statement: The data presented in this study are available on request.

Conflicts of Interest: It is declared that there is no conflicts of interest related to the authors of this article.

Reference

[1] Jose A, Lee S-M. Environmental reporting of global corporations: A content analysis based on website disclosures. Journal of business ethics. 2007; 72: 307-321.

[2] Adrian W. The big picture. Environment Business, 2007; 125: 15.

[3] Hummel K, Schlick C, policy p. The relationship between sustainability performance and sustainability disclosure – Reconciling voluntary disclosure theory and legitimacy theory. Journal of accounting. 2016; 35(5): 455-76.

[4] Sun PY. Stock Price Response to Voluntary Carbon Information Disclosure: The Moderating Effect of Carbon Risk Management. Korean Business Education Review, 2013; 28(2): 57-76.

[5] Zhang CP, Xiao X. International carbon information disclosure and its enlightenment to China. Finance and Finance, 2010; (03): 77-80.

[6] Zheng Y, Wu J, Fan TZ. Discussion on the accounting treatment of enterprise carbon emission right under the background of carbon peak and carbon neutrality. Chinese Certified Public Accountants, 2021; (09): 78-80.

[7] Chen H, Wang HY, Jing X. Carbon information disclosure of Chinese enterprises: research on content definition, measurement method and current situation. Accounting Research, 2013; (12): 18-24 + 96.

[8] Reinhardt F. Market failure and the environmental policies of firms: Economic rationales for "beyond compliance" behavior. Journal of industrial ecology. 1999; 3(1): 9-21.

[9] Marlene, Plumlee, Darrell, Brown, Rachel, M., et al. Voluntary environmental disclosure quality and firm value: Further evidence. Journal of Accounting Public Policy. 2015.

[10] Siddique A, Akhtaruzzaman, Rashid A, Hammami H. Carbon Disclosure, Carbon Performance and Financial Performance: International Evidence. Political Economy - Development: Environment Journal. 2021.

[11] Liesen A, Figge F, Hoepner AG, Patten DM. Climate Change and Asset Prices: Are Corporate Carbon Disclosure and Performance Priced Appropriately? Journal of Business Finance Accounting. 2017; 44(1-2): 35-62.

[12] Li L, Liu Q, Wang J, Hong X. Carbon Information Disclosure, Marketization, and Cost of Equity Financing. International Journal of Environmental Research Public Health. 2019; 16(1).

[13] Alsaifi K, Elnahass M, Salama A. Market responses to firms' voluntary carbon disclosure: Empirical evidence from the United Kingdom. Journal of Cleaner Production. 2020; 262:121377.

[14] Luo L, Lan YC, Tang Q. Corporate Incentives to Disclose Carbon Information: Evidence from the CDP Global 500 Report. Journal of International Financial Management Accounting. 2012; 23(2):93-120.

[15] Chen SY, Zhang JP, Liu CL. Environmental regulation, financing constraints, and enterprise pollution reduction —— Evidence from the adjustment of pollution discharge fee standard. Financial Research, 2021; (9): 51-71.

[16] Luo LL, Tang Q. Does national culture influence corporate carbon disclosure propensity. Journal of International Accounting Research. 2016; 15: 17-47.

[17] Li WJ. Ownership type, political rent-seeking, and CSR report: an analytical framework. Accounting Research, 2012; (1): 81-88 + 97.

[18] He Y, Tang QL, Wang Kaitian. Carbon information disclosure, carbon performance, and capital costs. Accounting Research, 2014; (1): 79-86 + 95.

[19] Hollindale J, Kent P, Routledge J, Chapple L. Women on boards and greenhouse gas emission disclosures. Accounting Finance. 2019; 59(1).

[20] Xu AL. Review on carbon Accounting Research for Enterprises. Contemporary Finance, 2014; (08): 111-120.

[21] Wang S, Wang H, Wang J, et al. Does environmental information disclosure contribute to improve firm financial performance? An examination of the underlying mechanism. Science of The Total Environment,2020; 714: 136855.

[22] Downar B, Ernstberger J, Reichelstein S, Schwenen S, Zaklan A. The impact of carbon disclosure mandates on emissions and financial operating performance. Review of Accounting Studies.1-39.

[23] Velte P, Stawinoga M, Lueg R. Carbon performance and disclosure: A systematic review of governance-related determinants and financial consequences. Journal of Cleaner Production. 2020; 254: 120063.

[24] Chen Z, Xie G.ESG disclosure and financial performance: Moderating role of ESG investors. International Review of Financial Analysis. 2022; 83: 102291.

[25] Hahn R, Reimsbach D, Schiemann F. Organizations, Climate Change, and Transparency: Reviewing the Literature on Carbon Disclosure. Social Science Electronic Publishing. 2015; 28(1): 80-102.

[26] Uyar A, Karaman AS, Kilic M. Is corporate social responsibility reporting a tool of signaling or greenwashing? Evidence from the worldwide logistics sector. Journal of Cleaner Production. 2020; 253: 119997.

[27] Peloza J. The Challenge of Measuring Financial Impacts From Investments in Corporate Social Performance. Journal of Management.2009; 35(6): 1518-1541.

[28] Brown N, Deegan C. The public disclosure of environmental performance information—a dual test of media agenda setting theory and legitimacy theory. Accounting Business Research. 1998; 29(1): 21-41.

[29] Fontana S, D'Amico E, Coluccia D, Solimene S. Does environmental performance affect companies' environmental disclosure? Measuring Business Excellence. 2015; 19(3): 42-57.

[30] Gray, Rob, Kouhy, Reza, Lavers, Simon. Corporate social and environmental reporting: a review of the literature and a longitudinal study of UK disclosure. Accounting Auditing Accountability Journal. 1995.

[31] Li L, Liu QQ, Tang DL. Carbon performance, quality of carbon information disclosure and equity financing cost. Management Review. 2019; 31 (1): 221-235.

[32] Zhang J, Ding R, Lu LW. The impact of carbon information disclosure and carbon performance on financial performance in the energy industry. Enterprise Technology Development. 2017; 36 (5): 96-97.

[33] Porter ME, Linde CVD. Toward a New Conception of the Environment-Competitiveness Relationship. Journal of Economic Perspectives. 1995;9(4):97-118.

[34] Ahern KR, Daminelli D, Fracassi C. Lost in translation? The effect of cultural values on mergers around the world. Journal of Financial Economics. 2015;117(1):165-89.

[35] Wu W, Firth M, Rui OM. Trust and the provision of trade credit. Journal of Banking Finance. 2014;39(2):146-59.

[36] A KH, B CS. The relationship between sustainability performance and sustainability disclosure – Reconciling voluntary disclosure theory and legitimacy theory. Journal of Accounting Public Policy. 2016;35(5):455-476.

[37] Milgrom P. Good Nevs and Bad News: Representation Theorems and Applications. Bell Journal of Economics. 1981; 2(12):380-391.

[38] Athanasouli D, Goujard A. Corruption and Management Practices, Firm Level Evidence. Journal of Comparative Economics. 2015;43(4):1014-34.

[39] Cai H, Fang H, Xu, Food LCJ, Machinery. Eat, Drink, Firms and Government: An Investigation of Corruption from Entertainment and Travel Costs of Chinese Firms. 2011;54(1):55-78.

[40] Tullock G. Problems of voting. Public Choice. 2005; 123(1/2): 49-58.

[41] Sims, G.C.. Rethinking the Political Power of American Business: The Role of Corporate Social Responsibility. Stanford University, 2003.

[42] Godfrey PC. The Relationship Between Corporate Philanthropy And Shareholder Wealth: A Risk Management Perspective. Academy of Management Review. 2005;30(4):777-798.

[43] Richardson AJ, Welker M, Hutchinson IR. Managing Capital Market Reactions to Corporate Social Resposibility. International Journal of Management Reviews. 2010;1(1):17-43.

[44] Pan AL, Liu X, Qiu JL, Shen Y. Whether the green mergers and acquisitions under the pressure of the media can promote the substantial transformation of heavily polluting enterprises. Industrial Economy of China. 2019; (2): 174-192.

[45] Wen SB, Zhou GL. The influence mechanism of enterprise carbon information disclosure on financial performance —— The "inverted U-shaped" regulatory effect of media governance. Management Review. 2017; 29 (11): 183-195.

[46] Shen HT, Feng J. Supervision by public opinion, government supervision and enterprise environmental information disclosur. Accounting Research. 2012; (02): 72-78 + 97.

[47] Song XH, Jiang X, Han JJ, et al. Research on the value effect of enterprise carbon information disclosure —— The regulatory effect based on financing constraints. Accounting Research. 2019; (12): 78-84.

[48] Clarkson PM, Li Y, Richardson GD, Vasvari FP. Revisiting the relation between environmental performance and environmental disclosure: An empirical analysis. Accounting Organizations Society. 2008; 33(4-5): 303-27.

[49] Dennis, M., Patten. The relation between environmental performance and environmental disclosure: a research note. Accounting, Organizations Society. 2002.

[50] Iatridis GE. Environmental disclosure quality: Evidence on environmental performance, corporate governance and value relevance. Emerging Markets Review. 2013; 14(3):55 – 75.

[51] Shira C, Igor K, Gaizka O, et al. Institutional Investors, Climate Disclosure, and Carbon Emissions. Journal of Accounting and Economics. 2023; (76): 2-3.

[52] Li, WJ, Lu, XY. Do institutional investors pay attention to firms' environmental performance? --Empirical Evidence from Listed Companies in Heavily Polluted Industries in China. Financial Research. 2015; (12): 97-112.

[53] Xu AL, Chen JL. Can institutional investors curb overinvestment —— Based on the test of the intermediary effect of free cash flow. Monthly Accounting. 2017; (18): 3-10.

[54] Verrecchia, Robert, E. Essays on disclosure. Journal of Accounting Economics. 2001; 32:97-180.

[55] Cui YG, Zhou C. Research on the status quo of carbon emission right trading and carbon accounting in The Beijing-Tianjin-Hebei Region. Accounting Research. 2017; (07): 3-10 + 96.

[56] Hasan I, Hoi CK, Wu Q, Zhang H. Social Capital and Debt Contracting: Evidence from Bank Loans and Public Bonds. Social Science Electronic Publishing. 2015;52(03):1-31.

[57] Gu LL, Guo JL, Wang HY. Corporate social responsibility, financing constraints and corporate Financialization. Financial Research. 2020; (02): 109-127.

[58] Hu XY. Political identity of private entrepreneurs and financing convenience of private enterprises- -Take the top 100 Private enterprises in Zhejiang Province as an example. Management World. 2006; (5): 107-113.

[59] Li GZ, Liu L. Debt financing costs and private credit discrimination. Financial Research. 2009; (12): 137-150.

[60] Shen HT, You JX, Liu JH. Environmental protection verification, environmental information disclosure and equity capital cost of refinancing. Financial Research. 2010; (12): 159-172.

[61] Marianne B, Esther D, Sendhil M. How Much Should We Trust Differences-In-Differences Estimates? Quarterly Journal of Economics. 2003; (1): 249-275.

[62] Sun LY, Miao CL, Yang L. Ecological-economic efficiency evaluation of green technology innovation in strategic emerging industries based on entropy weighted TOPSIS method. Ecological Indicators. 2017; 73: 554-558.
